# Repair versus reconstruction for proximal anterior cruciate ligament tears: a study protocol for a prospective multicenter randomized controlled trial

**DOI:** 10.1186/s12891-021-04280-y

**Published:** 2021-04-30

**Authors:** Jelle P. van der List, Harmen D. Vermeijden, Inger N. Sierevelt, Maarten V. Rademakers, Mark L. M. Falke, Gijs T. T. Helmerhorst, Roy A. G. Hoogeslag, Wybren A. van der Wal, Arthur van Noort, Gino M. M. J. Kerkhoffs

**Affiliations:** 1 Department of Orthopaedic Surgery, Amsterdam UMC, University of Amsterdam, Amsterdam, The Netherlands; 2 Department of Orthopaedic Surgery, Spaarne Gasthuis, Hoofddorp, The Netherlands; 3 Orthopaedic Trauma Service, Department of Orthopaedic Surgery, Hospital for Special Surgery, New York, NY USA; 4 Department of Orthopaedic Surgery, Flevoziekenhuis, Almere, The Netherlands; 5 Department of Orthopaedic Surgery, Centre for Orthopaedic Surgery OCON, Hengelo, The Netherlands; 6 Department of Orthopaedic Surgery, Ziekenhuis Gelderse Vallei, Ede, The Netherlands; 7 Amsterdam UMC, University of Amsterdam, Academic Center for Evidence based Sports medicine (ACES), Amsterdam, The Netherlands; 8 Amsterdam UMC, Amsterdam Collaboration on Health & Safety in Sports (ACHSS), University of Amsterdam and Vrije Universiteit Amsterdam IOC Research Center, Amsterdam, The Netherlands

**Keywords:** Anterior cruciate ligament, Anterior cruciate ligament reconstruction, Anterior cruciate ligament repair, Primary repair, Proximal tear, Knee injury, Suture repair, Remnant, Ligament preservation

## Abstract

**Background:**

For active patients with a tear of the anterior cruciate ligament (ACL) who would like to return to active level of sports, the current surgical gold standard is reconstruction of the ACL. Recently, there has been renewed interest in repairing the ACL in selected patients with a proximally torn ligament. Repair of the ligament has (potential) advantages over reconstruction of the ligament such as decreased surgical morbidity, faster return of range of motion, and potentially decreased awareness of the knee. Studies comparing both treatments in a prospective randomized method are currently lacking.

**Methods:**

This study is a multicenter prospective block randomized controlled trial. A total of 74 patients with acute proximal isolated ACL tears will be assigned in a 1:1 allocation ratio to either (I) ACL repair using cortical button fixation and additional suture augmentation or (II) ACL reconstruction using an all-inside autologous hamstring graft technique. The primary objective is to assess if ACL repair is non-inferior to ACL reconstruction regarding the subjective International Knee Documentation Committee (IKDC) score at two-years postoperatively. The secondary objectives are to assess if ACL repair is non-inferior with regards to (I) other patient-reported outcomes measures (i.e. Knee Injury and Osteoarthritis Outcome Score, Lysholm score, Forgotten Joint Score, patient satisfaction and pain), (II) objective outcome measures (i.e. failure of repair or graft defined as rerupture or symptomatic instability, reoperation, contralateral injury, and stability using the objective IKDC score and Rollimeter/KT-2000), (III) return to sports assessed by Tegner activity score and the ACL-Return to Sports Index at two-year follow-up, and (IV) long-term osteoarthritis at 10-year follow-up.

**Discussion:**

Over the last decade there has been a resurgence of interest in repair of proximally torn ACLs. Several cohort studies have shown encouraging short-term and mid-term results using these techniques, but prospective randomized studies are lacking. Therefore, this randomized controlled trial has been designed to assess whether ACL repair is at least equivalent to the current gold standard of ACL reconstruction in both subjective and objective outcome scores.

**Trial registration:**

Registered at Netherlands Trial Register (NL9072) on 25th of November 2020.

## Background

### Historical overview of ACL repair

The first documented surgical treatment of an anterior cruciate ligament (ACL) injury consisted of open repair in 1895 when Mayo Robson repaired a proximally avulsed ACL and posterior cruciate ligament back to the femur in a 41-year old male with good outcomes at six-year follow-up [1]. In the twentieth century, Ivar Palmar [2, 3] and Don O’Donoghue [4, 5] reported on open primary repair as a treatment of ACL injuries, and in the early 1970s open primary repair became a popular treatment for ACL injuries [6–9].

Feagin and Curl were the first to present the outcomes of open repair in 1972 and noted good outcomes at short-term follow-up [8]. A few years later in 1976, however, they noted a deterioration of outcomes at mid-term follow-up in their cohort [10]. Similarly, several other surgeons and researchers noted good short-term [11–16] but disappointing mid-term outcomes [17–21]. With these disappointing results and the promising outcomes of ACL reconstruction, several (randomized) prospective studies were started in the 1980s comparing open ACL repair with open ACL reconstruction [19, 22–24]. These prospective studies noted more reliable outcomes with ACL reconstruction when compared to ACL repair, which ultimately led to an abandonment of open ACL repair and to the current gold standard of ACL reconstruction for all patients [9].

In 1991, Sherman et al. were the first analyzing the disappointing mid-term outcomes of open ACL repair by performing an extensive subgroup analysis [21]. The authors found that a trend towards better outcomes in patients with proximal avulsion type tears and good tissue quality when compared to patients with midsubstance tears and/or tears with poor tissue quality. Unfortunately, the inclusion of the aforementioned prospective trials was already completed before the study by Sherman et al. was published, and thus the prospective trials contained all tear types including patients that might not have been ideal candidates for ACL repair (i.e., those with midsubstance tears or tears with poor tissue quality).

When critically reviewing the historical literature, and bearing in mind these findings by Sherman et al., it can be noted that the results of open repair of proximal ACL tears were indeed better. A recent systematic review of all historical studies on open repair noted that outcomes of open repair of proximal ACL tears showed 83 to 90% clinical stability, 80% return to sports, 79% good to excellent Lysholm score and 86% satisfaction in 539 patients in 11 studies [25]. These findings indicate that ACL repair may have been prematurely abandoned for all tear types and perhaps may be a good treatment option for patients with proximal tears. Furthermore, outcomes of ACL repair can be expected to improve when benefiting from modern development, such as arthroscopy (instead of open repair) and modern rehabilitation (instead of casting and immobilization).

### Rationale for ACL repair

The rationale behind better outcomes of ACL repair of proximal tears compared to midsubstance tears is that better vascularity is present at the proximal end of the ligament [26] and, as a result, proximal tears have healing potential for reattachment that is similar to medial collateral ligament (MCL) tears [27]. The reason for the continued pursuit of repair as a treatment of ACL injuries can also be explained by the potential advantages of repair over reconstruction. With ACL repair, the native tissue can be preserved along with proprioception which may provide patients with a more normal feeling of the knee compared to ACL reconstruction [28, 29]. Also, ACL repair is a less invasive surgery when compared to ACL reconstruction as no (or only small) tunnels need to be drilled and no graft tissues need to be harvested, leading to lower surgical morbidity [30–33], faster return of range of motion and fewer complications [34]. Furthermore, in case of failure of both treatments, revision surgery following primary repair is expected to be similar to primary reconstruction (no or only small tunnels have been drilled or grafts harvested), whereas revision of reconstruction surgery can be complicated by tunnel malpositioning or widening and pre-existing hardware and is associated with inferior outcomes compared to primary ACL reconstruction [35–37].

### Recent literature on ACL repair

With the recognized relevance of tear location in ACL repair and the potential advantages of this treatment, several surgeons and researchers have pursued the concept of ACL repair of proximal tears [38–47]. Most of these studies were retrospective small case series reporting good short-term outcomes with an overall reported failure rates of 6 to 9%, reoperation rates of 0 to 4% and patient-reported outcome measures (PROMs) > 85% of the maximum score [48]. Three studies have also shown that the good outcomes are maintained at mid-term follow-up [44, 45, 49]. One prospective study has compared the outcomes of repair (*n* = 20) versus reconstruction (*n* = 20) in patients with proximal tears and reported similar outcomes regarding functional outcomes, failure rates and laxity examination [46]. However, no randomized studies or studies with sufficient number of patients to assess differences between the treatments have been performed, and a recent systematic review also concluded higher-level evidence studies for ACL repair are currently lacking [48]. Recent studies have also suggested that primary repair with suture augmentation results in lower failure rates when compared to primary repair without suture augmentation [42, 48].

The current surgical gold standard of treating ACL injuries is ACL reconstruction using autograft tissue of either hamstring tendons, patellar tendon or quadriceps tendon. As for all new surgical techniques, the outcomes of arthroscopic ACL repair need to be compared to the current gold standard in order to assess whether this treatment can be used for standard patient care. Therefore, a randomized controlled trial (RCT) comparing ACL repair with ACL reconstruction is needed. The ACL study group of the Dutch Arthroscopy Association also recently declared that “*the application of ACL repair could be considered in a medial ethical committee-approved study until there is high-grade and long-term evidence regarding the efficacy of modern-day ACL repair*.”

### Goal and hypotheses

The goal of this multicenter non-inferior prospective randomized controlled trial is therefore to compare the outcomes of arthroscopic ACL repair with suture augmentation to ACL reconstruction for patients with proximal tears in a 1:1 allocation ratio. The primary outcome is the subjective International Knee Documentation Committee (IKDC) score and the secondary outcomes are other patient-reported outcomes, objective outcomes and return to sports. It is hypothesized that patients following ACL repair with suture augmentation have non-inferior primary and secondary outcomes when compared to ACL reconstruction due to the less invasive surgery.

## Methods

This study and manuscript have been designed in accordance to the Standard Protocol Items: Recommendations for Interventional Trials (SPIRIT) guidelines.

### Study design

This study is a multicenter prospective RCT with randomization into two treatment arms: (I) arthroscopic ACL repair with suture augmentation and (II) arthroscopic ACL reconstruction surgery. This study is a non-inferiority study with the hypothesis that arthroscopic ACL repair is non-inferior to (equivalent or better than) arthroscopic ACL reconstruction. All patients with proximal tears will be randomized during the operation into one of these treatment arms and will be followed up to 10-years postoperatively.

### Study sample

**Fig. 1:**
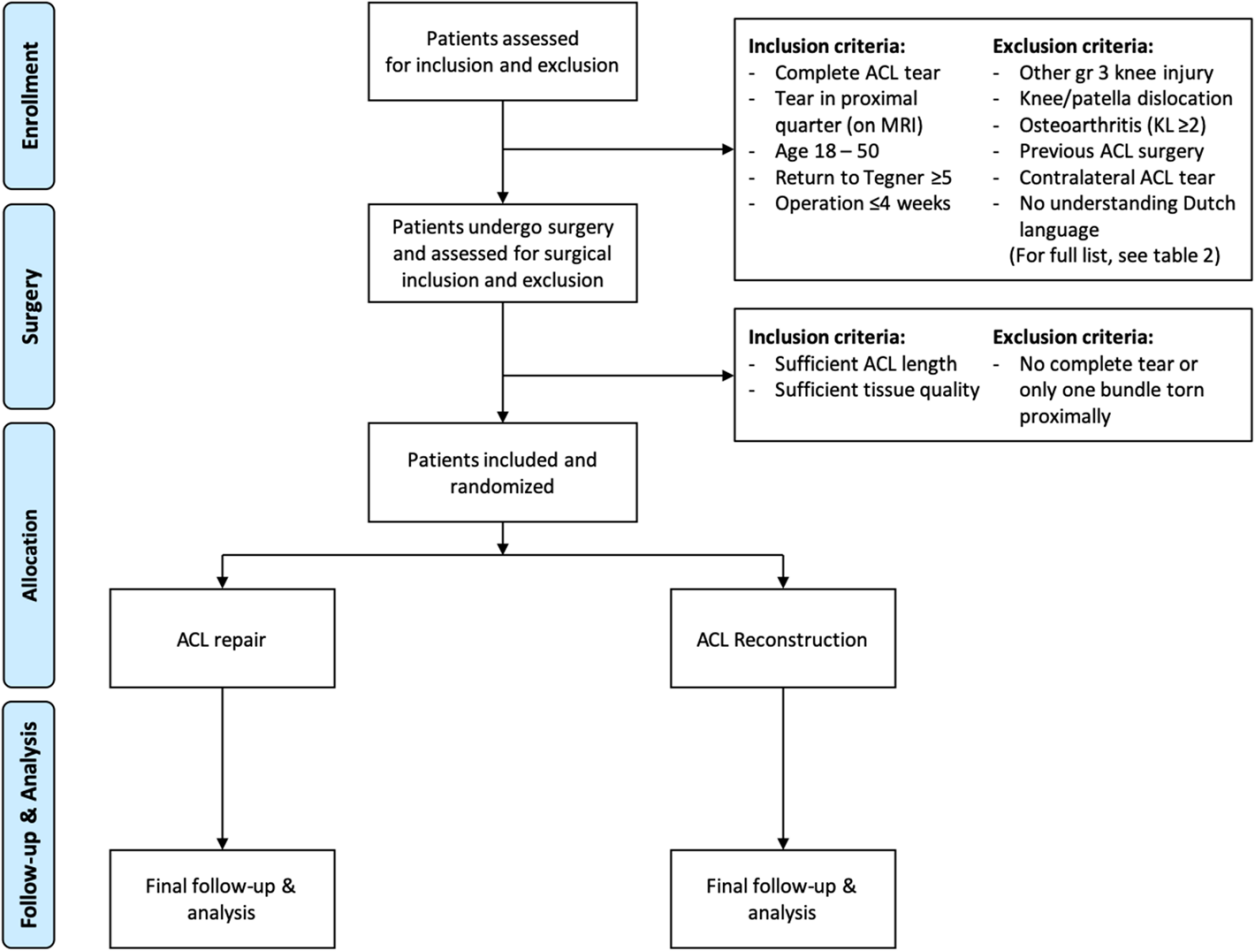
Flowchart of the REPAIR-trial

Potential candidates will be selected from five participating orthopaedic surgery departments, of which one is an academic hospital, three are teaching hospitals and one is a private hospital. Inclusion and exclusion criteria for participation in the study are displayed in Table 1. In general, potential inclusion involves all patients with acute, isolated, complete, proximal ACL tears that have a desire to return to pre-injury activities and exclusion involves all concomitant ligamentous and osteoarthritic injuries and skeletally immature patients. A flowchart of the study is shown in Fig. 1. Patients can withdraw their participation in this study at any time point, at which their data will be deleted.

**Table 1:** Inclusion and exclusion criteria for participating in this trial

Inclusion criteria	Exclusion criteria
**Pre-operative**	
Complete primary ACL tear on physical examination and MRI	Complete ipsilateral concomitant knee ligament injury requiring surgery
Tear in proximal quarter on MRI [50, 51]	Concomitant ipsilateral knee dislocation or patellar dislocation
Age 18 – 50 years [22, 52]	Osteoarthritis KL grade ≥ 2
Preinjury Tegner level ≥ 5 & desired Tegner level ≥ 5 [53]	Previous ipsilateral ACL reconstruction/repair
Operation within 4 weeks of injury [54]	Intra-articular corticosteroids 6 months prior
	No understanding of Dutch language or not capable of understanding the study and participation
	No preoperative flexion of 90 degrees
	Grade 3 pivot shift indicating gross ligament instability that requires additional procedures
	Gross lower leg malalignment requiring bony osteotomies
	Muscular, neurological or vascular diseases that influence rehabilitation or surgery
	Prolonged use medication use of prednison or cytostatics
	Pregnancy during injury or surgery
	Osteoporosis that influence rehabilitation or surgery
**Intra-operative**	
Sufficient tissue length for retensioning to femoral insertion	No complete tear at arthroscopy or only one bundle (AM or PL) with proximal tear
Sufficient tissue quality to withhold sutures	Grade 3 or grade 4 cartilage lesions
ACL indicates anterior cruciate ligament; MCL, medial collateral ligament; LCL, lateral collateral ligament; PCL, posterior cruciate ligament; PLC, posterolateral corner.

### Randomization

All patients will be consented preoperatively for the study. Patients are taken into the operating room, general or epidural anesthesia is induced, and the leg is prepped and draped for standard arthroscopic knee surgery with a tourniquet high at the upper thigh. Then standard anteromedial and anterolateral portals are created, and the knee is assessed for cartilage, meniscus and ligamentous injuries. After cartilage and meniscus injuries are addressed, the tear type of the ACL and eligibility for this study is assessed. First, it should be confirmed whether a proximal tear is present (i.e., whether the distal remnant of the ACL is of sufficient length to be reattached to the anatomical femoral footprint of the ACL) and whether sufficient tissue quality is present (i.e., whether the ligament remnant is of sufficient quality to withhold suture passage and can be tensioned towards the femur).

**Fig. 2:**
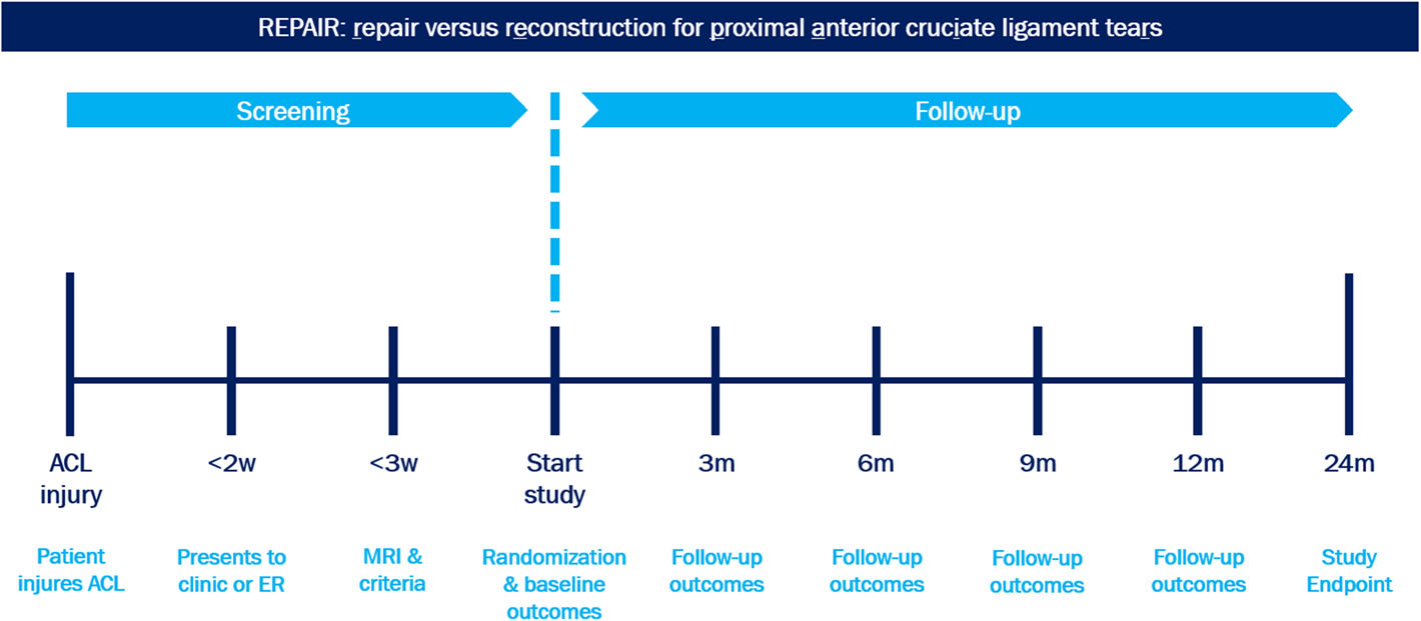
Timeline for patients in the REPAIR-trial

If these conditions are present, patients are randomized between both treatment arms, and if these conditions are not present, the patient is excluded, and standard ACL reconstruction will be performed. A computer block randomization of 10 patients per block will be done digitally prior to the study, and the allocation concealment is performed by sequentially numbered, opaque, sealed envelopes containing the name of the procedure in a randomized order. The envelopes are placed in the operating room and opened when the surgeon deems the ACL tear eligible for the study. A participant timeline is shown in Fig. 2.

### Surgical techniques

Prior to the start of the trial, a cadaver session will be held in order to standardize the technique of ACL repair and ACL reconstruction for all surgeons and to minimize the learning curve. All surgeons have extensive experience with ACL reconstruction and two out of five participating centers have experience with ACL repair.

The surgical technique of arthroscopic ACL repair has been more extensively described in the literature [39, 43, 55]. In brief, first the native torn ACL will be sutured with a loop using FiberWire sutures and advanced with one to two passes, so that the sutures exit the avulsed ligament towards the femur. Then, a small tunnel will be drilled from the native femoral insertion towards the lateral epicondyle using an ACL drill guide. The sutures will be passed through a TightRope button along with an additional FiberTape. The sutures and TightRope will be passed through the femoral tunnel and the button will be flipped. Then, a small tunnel will be drilled through the tibia from the anteromedial cortex towards the anterior part of the tibial footprint, and the FiberTape will be channeled through the tibial tunnel and, after cycling the knee, the FiberTape is fixed into the anteromedial cortex using a suture anchor at full extension. Finally, the repair sutures will be tensioned and tied in order to reapproximate the ACL towards the femoral footprint at 90° flexion.

For ACL reconstruction, a standard all-inside autograft hamstring tendon anatomic reconstruction technique is used [56, 57]. First, autologous hamstrings (semitendinosus and gracilis tendon) are harvested to the preference of the surgeon and will be prepared for graft usage with a minimum graft diameter of 8 mm [58, 59]. Then, femoral and tibial sockets are independently drilled in retrograde fashion using a FlipCutter drill. The graft is placed into the sockets, the knee is cycled in order to achieve optimal tension of the graft, and the graft is then fixed at the femoral and tibial side using a cortical button.

### Rehabilitation

Both treatment arms undergo the same rehabilitation program and consists of a milestone-based program according to the Dutch national guidelines for rehabilitation following ACL reconstruction and consists of three phases [60–62]. The first phase focuses on controlling swelling, restoration of range of motion and return of quadriceps muscle control, and generally takes 4 to 8 weeks. The second phase focuses on resuming light sporting activities and work without symptoms, and phase three focuses on full return to sports activities and heavy work. In case of meniscus repair, the first 6 weeks patients are partial weight bearing, range of motion is restricted to 0-90° and patients are not allowed deep bending or squatting for 4 months. Although the rehabilitation is milestone based and no strict time goals can be set, generally cycling on a stationary bike is allowed at 4-6 weeks, running at 10-12 weeks and return to sports and pivoting activities at a minimum of 9 months postoperatively.

### Blinding

Blinding for patients is not possible due to different scars, different postoperative radiographs and practical reasons. However, the data analysis will be performed in blinded fashion.

### Primary outcomes/endpoint (Table 2)

The primary outcome of this non-inferiority RCT is the subjective patient reported outcome (PROM) at two-year follow-up consisting of the subjective IKDC score [63] (Dutch validation [64]), as to a recent RCT on a similar topic [65, 66]. The primary endpoint is the subjective IKDC at two-years postoperatively. Patients will ultimately be followed for 10 years.

**Table 2:** This chart provides an overview of which outcomes are collected at the different follow-up visits

	Pre	3 mns	6 mns	9 mns	1 yr	2 yrs	5 yrs	10 yrs
**Primary outcomes**								
IKDC subjective	X	X	X	X	X	X	X	X
**Secondary outcomes**								
KOOS	X	X	X	X	X	X	X	X
Lysholm	X	X	X	X	X	X	X	X
Forgotten Joint Score	X	X	X	X	X	X	X	X
Satisfaction & pain	X	X	X	X	X	X	X	X
Failure		X	X	X	X	X	X	X
Reoperation		X	X	X	X	X	X	X
Contralateral injury	X	X	X	X	X	X	X	X
IKDC objective	X	X	X	X	X	X	X	X
KT-1000	X	X	X	X	X	X	X	X
Return to sports		X	X	X	X	X	X	X
Tegner score	X	X	X	X	X	X	X	X
ACL-RSI		X	X	X	X	X	X	X
Osteoarthritis (X-ray)	X							X
AE, SAE, SUSAR	X	X	X	X	X	X	X	X

*IKDC* indicates International Knee Documentation Committee, *KOOS* Knee Injury and Osteoarthritis Outcome Score, *AE* adverse events, *SAE* serious adverse event, *SUSAR* Suspected Unexpected Serious Adverse Reaction, *Pre* preoperatively, *mns* months, *yr(s)* year(s)

### Secondary outcomes (Table 2)

The secondary outcomes of this RCT are fourfold and consist of (I) other subjective outcomes, (II) objective outcomes, (III) return to sports, and (IV) long-term osteoarthritis.

Other collected PROMs for this study are the Knee Injury and Osteoarthritis Outcome Score (KOOS) [67] (Dutch validation [68]), Lysholm score [69] (Dutch validation [70]), and Forgotten Joint Score (FJS) [28] (Dutch validation [71]). Furthermore, patient satisfaction and pain scores are collected using a numeric rating scale (range 0 – 10).

The objective outcomes consist of failure of ACL repair/graft, reoperation, contralateral injury, and laxity. Failure is defined as a (traumatic) rerupture or symptomatic instability with activities. Reoperation is defined as any new operation on the same knee for any other reason than revision (e.g., symptomatic meniscus tear, hardware irritation, infection or stiffness/arthrofibrosis). Contralateral injury was defined as a complete ACL rupture of the contralateral ACL. Stability is defined as the laxity found with physical examination using the IKDC objective score form [72], which includes the Lachman, anterior drawer and pivot shift test, and side-to-side differences is assessed using KT-2000 or Rollimeter.

Return to sports is defined as (I) returning to sports, (II) returning to the same sport, and (III) returning to the preinjury level of sport. The preinjury and postoperative Tegner activity scale are also collected, which enables comparison with other studies [73] (Dutch validation [70]). Finally, confidence of return to sports and fear of reinjury are assessed using the ACL-Return to Sports Index (ACL-RSI) score [74] (Dutch validation [75]).

Osteoarthritis will be reviewed at 10-year follow-up. Radiographs of both knees will be performed, and the operated knee will be compared to (I) the contralateral knee if no operation occurred in that knee, and (II) the ipsilateral knee radiograph preoperatively. The Kellgren-Lawrence (KL) grade will be used to assess the incidence and grades of osteoarthritis.

### Sample size

The sample size calculation was based on the primary outcome of this study (subjective IKDC score), similar to another RCT design on this topic [65]. It has been shown that a difference of 8.8 points in the subjective IKDC score is the minimal clinically important difference (MCID) [76]. Using this non-inferiority limit of 8.8 points, and a standard deviation of 11 points [42, 65, 77] along with a two-sided alpha of 0.05, a power of 90%, and a lost-to-follow-up rate of 10%, a total of 37 patients in each group (74 patients in total) are needed to assess the primary outcome of this non-inferiority RCT. This sample size is also sufficient for the MCID of KOOS [78] and Lysholm score [79]. Given the recent studies that showed that 30-40% of the acute tears will have repairable proximal ACL tears [50, 80], we estimate that approximately 200 patients will be needed to be screened preoperatively to achieve the sample size of 74 patients [81].

### Statistical analysis

Both an intention to treat analysis and per protocol analysis will be performed for this non-inferiority study. Comparison of nominal variables between ACL repair and ACL reconstruction will be performed using two-by-two tables with Pearson’s Chi-square test or Fisher’s exact test (in case one of the cells is < 5). For comparison of continuous variables, first tests for normal distribution of values are performed and independent t-tests are used of normal distributed values and non-parametric t-tests are used for not-normally distributed values.

A mixed model analysis for repeated measures will be performed to assess differences between both groups. Furthermore, a multivariate regression analysis will be performed for the primary endpoint of IKDC at two-years follow-up in order to correct for potential confounders. Statistical analysis will be performed using SPSS version 25.0 (IBM Software, Armonk, NY, USA). All tests are two-sided and a *p*-value of < 0.05 is considered statistically significant.

## Discussion

This study reports on the study design of the REPAIR-trial (**R**epair versus r**E**construction for **P**roximal **A**nterior cruciate l**I**gament tea**R**s). Few studies have examined the outcomes of repair versus reconstruction with favorable outcomes for ACL reconstruction [22–24]. However, these studies were performed over 30 years ago and are limited by the fact that all tear types were repaired rather than only proximal tears and that repair was performed using an arthrotomy [9, 25, 82]. Recently, four RCT studies have been designed to assess the outcomes of ACL repair [65, 83–85] but these are either performed in midsubstance tears [65, 83], assess the outcomes of dynamic intraligamentary stabilization (DIS) versus ACL reconstruction [65, 83], repair versus DIS [84] or Bridge-Enhanced ACL Repair (BEAR) with reconstruction [85]. Our current RCT differs from these studies as only proximal tears will be treated rather than all tear types and as the ligament will be reattached to the femoral footprint in a minimally invasive way.

The renewed interest of repair of proximal tears can be explained by improved understanding of patient selection. Research has shown that proximal tears have a better vascularity compared to midsubstance tears [26] and therefore have excellent healing capacity by reattachment to the femoral wall which is similar to the healing capacity of MCL tears [27]. Both historical studies on open ACL repair [9, 25, 82] and more recent studies on repair with DIS (also known as Ligamys) have shown that the clinical outcomes are indeed better when repairing proximal tears. Two studies have shown failure rates of repair with DIS in midsubstance tears of 24% in all patients and 36% in competitive athletes with midsubstance tears [86, 87]. Our current study applies strict patient selection criteria of proximal tears and good tissue quality. As the length of distal remnant and possibility of repair can only be assessed intraoperatively, randomization in this study should perform during surgery after the surgeon has confirmed the possibility of repair. Consequently, patients will be consented that they might be excluded during surgery if a non-repairable tear is present, and these patients will undergo standard ACL reconstruction.

It should be noted that there is also a potential disadvantage of ACL repair. By performing ACL surgery in the early phase (since early surgery prevents ligament retraction and preserves tissue quality that is both needed for repair [4, 5, 88]), it is likely that too many ACL surgeries will be performed. Current day standards recommend that patients following ACL injury will be treated conservatively first as approximately half of the patient may be copers and do not need surgical intervention [53, 60, 89]. By performing surgery on all ACL injured patients, patients will undergo surgery while they might be copers and do not need surgery. This risk is minimized in this study by only including patients aged 18 – 50 and only patients that desire to return to sports. It would be best if it is known preoperatively which patients will not do well with conservative treatment and ultimately require ACL surgery, as this both increases the chance of performing ACL repair and as early reconstruction outcomes decreases the risk for meniscal and chondral damage [60] at longer follow-up when compared to delayed reconstruction.

Several studies have recently reported good short-term outcomes of arthroscopic ACL repair using different techniques: in some studies femoral fixation consisted of using two suture anchors [42, 44], one suture anchor (for both bundles) [40, 45, 46] or transosseous tunnels with or without cortical button fixation [39, 41, 43, 55, 90], and some studies used ACL repair without [40, 41, 45, 46] or with [39, 43, 55, 90] additional suture augmentation. For this study, femoral fixation will consist of cortical button fixation with additional suture augmentation (FiberTape) in order to protect the repair in the early phases of rehabilitation, becuase it has been suggested that additional suture augmentation leads to lower rerupture rates [42, 48].

This study has been designed to assess the outcomes following repair and reconstruction of proximal ACL tears. We hypothesize that the repair treatment is a good treatment for proximal tears as it has potential advantages over ACL reconstruction: the surgery is short and minimally invasive, it has a low complication rate, rehabilitation is easier, and in case ACL repair fails then primary reconstruction surgery can be performed. Non-inferiority of arthroscopic ACL repair compared to arthroscopic ACL reconstruction may lead to a treatment algorithm in which patients with proximal avulsion tears can be repaired in the acute setting whereas patients with midsubstance tears will undergo ACL reconstruction in either the acute or delayed setting [91, 92].

## Data Availability

Available at study publication.

## Abbreviations

ACL: Anterior Cruciate Ligament
ACL-RSI: Anterior Cruciate Ligament - Return to Sports Index
BEAR: Bridge-Enhanced ACL Repair
DIS: Dynamic Intraligamentary Stabilization
FJS: Forgotten Joint Score
IKDC: International Knee Documentation Committee
KOOS: Knee Injury and Osteoarthritis Outcome Score
MCID: Minimal Clinically Important Difference
MCL: Medial Collateral Ligament
PROM: Patient-Reported Outcome Measure
RCT: Randomized Controlled Trial
REPAIR-trial: **R**epair versus r**E**construction for **P**roximal **A**nterior cruciate l**I**gament tea**R**s trial
SPIRIT: Standard Protocol Items: Recommendations for Interventional Trials

## References

[1] Robson AW (1903). VI. Ruptured crucial ligaments and their repair by operation. Ann Surg.

[2] Palmer I. On the injuries to the ligaments of the knee joint. Acta Orthop Scand. 1938;53.

[3] Palmer I (2007). On the injuries to the ligaments of the knee joint: a clinical study. 1938. Clin Orthop Relat Res.

[4] O'Donoghue DH (1955). An analysis of end results of surgical treatment of major injuries to the ligaments of the knee. J Bone Joint Surg Am.

[5] O’Donoghue DH (1950). Surgical treatment of fresh injuries to the major ligaments of the knee. J Bone Joint Surg Am.

[6] van der List WP (1964). De operatieve behandeling van de bandverscheuringen van de knie. Ned Tijdschr Geneeskd.

[7] Liljedahl SO, Lindvall N, Wetterfors J (1965). Early diagnosis and treatment of acute ruptures of the anterior cruciate ligament; a clinical and arthrographic study of forty-eight cases. J Bone Joint Surg Am.

[8] Feagin JA, Abbott HG, Rokous JR (1972). The isolated tear of the anterior cruciate ligament. J Bone Joint Surg Am.

[9] van der List JP, DiFelice GS (2017). Primary repair of the anterior cruciate ligament: a paradigm shift. Surgeon.

[10] Feagin JA, Curl WW (1976). Isolated tear of the anterior cruciate ligament: 5-year follow-up study. Am J Sports Med.

[11] Cabitza P, Colombo A, Verdoia C (1978). Follow-up of results obtained with O'Donoghue's technique in the repair of recent lesions of the anterior cruciate ligament. Minerva Ortop.

[12] Nixon JE (1980). Acute injuries of the anterior cruciate ligament of the knee: primary repair. Bull N Y Acad Med.

[13] Marshall JL, Warren RF, Wickiewicz TL (1982). Primary surgical treatment of anterior cruciate ligament lesions. Am J Sports Med.

[14] Warren RF (1983). Primary repair of the anterior cruciate ligament. Clin Orthop Relat Res.

[15] Marcacci M, Spinelli M, Chiellini F, Buccolieri V (1985). Notes on 53 cases of immediate suture of acute lesions of the anterior cruciate ligament. Ital J Orthop Traumatol.

[16] Sherman MF, Bonamo JR (1988). Primary repair of the anterior cruciate ligament. Clin Sports Med.

[17] Odensten M, Lysholm J, Gillquist J (1984). Suture of fresh ruptures of the anterior cruciate ligament. A 5-year follow-up. Acta Orthop Scand.

[18] Engebretsen L, Benum P, Sundalsvoll S (1989). Primary suture of the anterior cruciate ligament a 6-year follow-up of 74 cases. Acta Orthop Scand.

[19] Jonsson T, Peterson L, Renstrom P (1990). Anterior cruciate ligament repair with and without augmentation. A prospective 7-year study of 51 patients. Acta Orthop Scand.

[20] Kaplan N, Wickiewicz TL, Warren RF (1990). Primary surgical treatment of anterior cruciate ligament ruptures. A long-term follow-up study. Am J Sports Med.

[21] Sherman MF, Lieber L, Bonamo JR, Podesta L, Reiter I (1991). The long-term followup of primary anterior cruciate ligament repair. Defining a rationale for augmentation. Am J Sports Med.

[22] Engebretsen L, Benum P, Fasting O, Molster A, Strand T (1990). A prospective, randomized study of three surgical techniques for treatment of acute ruptures of the anterior cruciate ligament. Am J Sports Med.

[23] Grontvedt T, Engebretsen L (1995). Comparison between two techniques for surgical repair of the acutely torn anterior cruciate ligament. A prospective, randomized follow-up study of 48 patients. Scand J Med Sci Sports.

[24] Grontvedt T, Engebretsen L, Benum P, Fasting O, Molster A, Strand T (1996). A prospective, randomized study of three operations for acute rupture of the anterior cruciate ligament. Five-year follow-up of one hundred and thirty-one patients. J Bone Joint Surg Am.

[25] van der List JP, DiFelice GS (2017). Role of tear location on outcomes of open primary repair of the anterior cruciate ligament: a systematic review of historical studies. Knee.

[26] Toy BJ, Yeasting RA, Morse DE, McCann P (1995). Arterial supply to the human anterior cruciate ligament. J Athl Train.

[27] Nguyen DT, Ramwadhdoebe TH, van der Hart CP, Blankevoort L, Tak PP, van Dijk CN (2014). Intrinsic healing response of the human anterior cruciate ligament: an histological study of reattached ACL remnants. J Orthop Res.

[28] Behrend H, Zdravkovic V, Giesinger JM, Giesinger K (2017). Joint awareness after ACL reconstruction: patient-reported outcomes measured with the forgotten joint Score-12. Knee Surg Sports Traumatol Arthrosc.

[29] Vermeijden HD, van der List JP, O'Brien R, DiFelice GS (2020). Patients forget about their operated knee more following arthroscopic primary repair of the anterior cruciate ligament than following reconstruction. Arthroscopy.

[30] Murray MM (2009). Current status and potential of primary ACL repair. Clin Sports Med.

[31] Adachi N, Ochi M, Uchio Y, Iwasa J, Ryoke K, Kuriwaka M (2002). Mechanoreceptors in the anterior cruciate ligament contribute to the joint position sense. Acta Orthop Scand.

[32] Gao F, Zhou J, He C, Ding J, Lou Z, Xie Q, Li H, Li F, Li G (2016). A morphologic and quantitative study of mechanoreceptors in the remnant stump of the human anterior cruciate ligament. Arthroscopy.

[33] Murray MM, Fleming BC, Badger GJ, Freiberger C, Henderson R, Barnett S, Kiapour A, Ecklund K, Proffen B, Team BT (2020). Bridge-enhanced anterior cruciate ligament repair is not inferior to autograft anterior cruciate ligament reconstruction at 2 years: results of a prospective randomized clinical trial. Am J Sports Med.

[34] van der List JP, DiFelice GS (2017). Range of motion and complications following primary repair versus reconstruction of the anterior cruciate ligament. Knee.

[35] Andriolo L, Filardo G, Kon E, Ricci M, Della Villa F, Della Villa S, Zaffagnini S, Marcacci M (2015). Revision anterior cruciate ligament reconstruction: clinical outcome and evidence for return to sport. Knee Surg Sports Traumatol Arthrosc.

[36] Arianjam A, Inacio MCS, Funahashi TT, Maletis GB: Analysis of 2019 Patients undergoing revision anterior cruciate ligament reconstruction from a community- based registry. Am J Sports Med 2017;45:1574–1580. 10.1177/0363546517700882 28426243

[37] van der List JP, Vermeijden HD, O’Brien R, DiFelice GS (2019). Anterior cruciate ligament reconstruction following failed primary repair: surgical technique and a report of three cases. Minerva Ortop Traumatol.

[38] DiFelice GS, Villegas C, Taylor SA (2015). Anterior cruciate ligament preservation: early results of a novel arthroscopic technique for suture anchor primary anterior cruciate ligament repair. Arthroscopy.

[39] Smith JO, Yasen SK, Palmer HC, Lord BR, Britton EM, Wilson AJ (2016). Paediatric ACL repair reinforced with temporary internal bracing. Knee Surg Sports Traumatol Arthrosc.

[40] Bigoni M, Gaddi D, Gorla M, Munegato D, Pungitore M, Piatti M, Turati M (2017). Arthroscopic anterior cruciate ligament repair for proximal anterior cruciate ligament tears in skeletally immature patients: surgical technique and preliminary results. Knee.

[41] Mukhopadhyay R, Shah N, Vakta R, Bhatt J (2018). ACL femoral avulsion repair using suture pull-out technique: a case series of thirteen patients. Chin J Traumatol.

[42] Jonkergouw A, van der List JP, DiFelice GS (2019). Arthroscopic primary repair of proximal anterior cruciate ligament tears: outcomes of the first 56 consecutive patients and the role of additional internal bracing. Knee Surg Sports Traumatol Arthrosc.

[43] Heusdens CHW, Hopper GP, Dossche L, Roelant E, Mackay GM (2019). Anterior cruciate ligament repair with independent suture tape reinforcement: a case series with 2-year follow-up. Knee Surg Sports Traumatol Arthrosc.

[44] DiFelice GS, van der List JP (2018). Clinical outcomes of arthroscopic primary repair of proximal anterior cruciate ligament tears are maintained at midterm follow-up. Arthroscopy.

[45] Hoffmann C, Friederichs J, von Ruden C, Schaller C, Buhren V, Moessmer C (2017). Primary single suture anchor re-fixation of anterior cruciate ligament proximal avulsion tears leads to good functional mid-term results: a preliminary study in 12 patients. J Orthop Surg Res.

[46] Achtnich A, Herbst E, Forkel P, Metzlaff S, Sprenker F, Imhoff AB, Petersen W (2016). Acute proximal anterior cruciate ligament tears: outcomes after arthroscopic suture anchor repair versus anatomic single-bundle reconstruction. Arthroscopy.

[47] Vermeijden HD, Yang XA, van der List JP, Difelice GS (2020). Role of age on success of arthroscopic primary repair of proximal anterior cruciate ligament tears. Arthroscopy.

[48] van der List JP, Vermeijden HD, Sierevelt IN, DiFelice GS, van Noort A, Kerkhoffs GMMJ (2020). Arthroscopic primary repair of proximal anterior cruciate ligament tears seems safe but higher level of evidence is needed: a systematic review and meta-analysis of recent literature. Knee Surg Sports Traumatol Arthrosc.

[49] Hopper GP, Aithie JMS, Jenkins JM, Wilson WT, Mackay GM. Satisfactory patient-reported outcomes at 5 years following primary repair with suture tape augmentation for proximal anterior cruciate ligament tears. Knee Surg Sports Traumatol Arthrosc. 2021. 10.1007/s00167-021-06485-z. 10.1007/s00167-021-06485-z PMC8800885 33582828

[50] van der List JP, DiFelice GS (2017). Preoperative magnetic resonance imaging predicts eligibility for arthroscopic primary anterior cruciate ligament repair. Knee Surg Sports Traumatol Arthrosc.

[51] Vermeijden HD, Cerniglia B, Mintz DN, Rademakers MV, Kerkhoffs G, van der List JP, et al. Distal remnant length can be measured reliably and predicts primary repair of proximal anterior cruciate ligament tears. Knee Surg Sports Traumatol Arthrosc. 2020. 10.1007/s00167-020-06312-x. 10.1007/s00167-020-06312-x 33057796

[52] Andersson C, Odensten M, Good L, Gillquist J (1989). Surgical or non-surgical treatment of acute rupture of the anterior cruciate ligament. A randomized study with long-term follow-up. J Bone Joint Surg Am.

[53] Frobell RB, Roos EM, Roos HP, Ranstam J, Lohmander LS (2010). A randomized trial of treatment for acute anterior cruciate ligament tears. N Engl J Med.

[54] van der List JP, Jonkergouw A, van Noort A, Kerkhoffs G, DiFelice GS (2019). Identifying candidates for arthroscopic primary repair of the anterior cruciate ligament: a case-control study. Knee.

[55] Mackay GM, Blyth MJ, Anthony I, Hopper GP, Ribbans WJ (2015). A review of ligament augmentation with the InternalBrace: the surgical principle is described for the lateral ankle ligament and ACL repair in particular, and a comprehensive review of other surgical applications and techniques is presented. Surg Technol Int.

[56] Connaughton AJ, Geeslin AG, Uggen CW (2017). All-inside ACL reconstruction: how does it compare to standard ACL reconstruction techniques?. J Orthop.

[57] Vinagre G, Kennedy NI, Chahla J, Cinque ME, Hussain ZB, Olesen ML, LaPrade RF (2017). Hamstring graft preparation techniques for anterior cruciate ligament reconstruction. Arthrosc Tech.

[58] Fu CW, Chen WC, Lu YC (2020). Is all-inside with suspensory cortical button fixation a superior technique for anterior cruciate ligament reconstruction surgery? A systematic review and meta-analysis. BMC Musculoskelet Disord.

[59] Tang SP, Wan KH, Lee RH, Wong KK, Wong KK. Influence of hamstring autograft diameter on graft failure rate in Chinese population after anterior cruciate ligament reconstruction. Asia Pac J Sports Med Arthrosc Rehabil Technol. 2020;22:45–8. 10.1016/j.asmart.2020.07.005 PMC7453058 32913712

[60] Meuffels DE, Poldervaart MT, Diercks RL, Fievez AW, Patt TW, Hart CP, Hammacher ER, Meer F, Goedhart EA, Lenssen AF (2012). Guideline on anterior cruciate ligament injury. Acta Orthop.

[61] van Melick N, van Cingel RE, Brooijmans F, Neeter C, van Tienen T, Hullegie W (2016). Nijhuis-van der Sanden MW: evidence-based clinical practice update: practice guidelines for anterior cruciate ligament rehabilitation based on a systematic review and multidisciplinary consensus. Br J Sports Med.

[62] Engelen-van Melick N, Hullegie W, Brooijmans F, Hendriks E, Neeter C, Van Tienen T, van Cingel R (2014). KNGF Evidence Statement; Revalidatie na voorste-kruisbandreconstructie.

[63] Irrgang JJ, Anderson AF, Boland AL, Harner CD, Kurosaka M, Neyret P, Richmond JC, Shelborne KD (2001). Development and validation of the international knee documentation committee subjective knee form. Am J Sports Med.

[64] Haverkamp D, Sierevelt IN, Breugem SJ, Lohuis K, Blankevoort L, van Dijk CN (2006). Translation and validation of the Dutch version of the international knee documentation committee subjective knee form. Am J Sports Med.

[65] Boer BC, Hoogeslag RAG, Brouwer RW, Demmer A, Huis RM (2018). Self-reported functional recovery after reconstruction versus repair in acute anterior cruciate ligament rupture (ROTOR): a randomized controlled clinical trial. BMC Musculoskelet Disord.

[66] Hoogeslag RAG, Brouwer RW, Boer BC, de Vries AJ, Huis RM (2019). Acute anterior cruciate ligament rupture: repair or reconstruction? Two-year results of a randomized controlled clinical trial. Am J Sports Med.

[67] Roos EM, Roos HP, Lohmander LS, Ekdahl C, Beynnon BD (1998). Knee injury and osteoarthritis outcome score (KOOS)--development of a self-administered outcome measure. J Orthop Sports Phys Ther.

[68] de Groot IB, Favejee MM, Reijman M, Verhaar JA, Terwee CB (2008). The Dutch version of the knee injury and osteoarthritis outcome score: a validation study. Health Qual Life Outcomes.

[69] Lysholm J, Gillquist J (1982). Evaluation of knee ligament surgery results with special emphasis on use of a scoring scale. Am J Sports Med.

[70] Eshuis R, Lentjes GW, Tegner Y, Wolterbeek N, Veen MR (2016). Dutch translation and cross-cultural adaptation of the Lysholm score and Tegner activity scale for patients with anterior cruciate ligament injuries. J Orthop Sports Phys Ther.

[71] Shadid MB, Vinken NS, Marting LN, Wolterbeek N (2016). The Dutch version of the forgotten joint score: test-retesting reliability and validation. Acta Orthop Belg.

[72] Hefti F, Muller W, Jakob RP, Staubli HU (1993). Evaluation of knee ligament injuries with the IKDC form. Knee Surg Sports Traumatol Arthrosc.

[73] Tegner Y, Lysholm J (1985). Rating systems in the evaluation of knee ligament injuries. Clin Orthop Relat Res.

[74] Webster KE, Feller JA, Lambros C (2008). Development and preliminary validation of a scale to measure the psychological impact of returning to sport following anterior cruciate ligament reconstruction surgery. Phys Ther Sport.

[75] Slagers AJ, Reininga IHF, van den Akker-Scheek I (2017). The Dutch language anterior cruciate ligament return to sport after injury scale (ACL-RSI) – validity and reliability. J Sports Sci.

[76] Irrgang JJ, Anderson AF, Boland AL, Harner CD, Neyret P, Richmond JC, Shelbourne KD (2006). Responsiveness of the international knee documentation committee subjective knee form. Am J Sports Med.

[77] Li YL, Ning GZ, Wu Q, Wu QL, Li Y, Hao Y, Feng SQ (2014). Single-bundle or double-bundle for anterior cruciate ligament reconstruction: a meta-analysis. Knee.

[78] Aga C, Risberg MA, Fagerland MW, Johansen S, Troan I, Heir S, Engebretsen L (2018). No difference in the KOOS quality of life subscore between anatomic double-bundle and anatomic single-bundle anterior cruciate ligament reconstruction of the knee: a prospective randomized controlled trial with 2 Years' follow-up. Am J Sports Med.

[79] Briggs KK, Lysholm J, Tegner Y, Rodkey WG, Kocher MS, Steadman JR (2009). The reliability, validity, and responsiveness of the Lysholm score and Tegner activity scale for anterior cruciate ligament injuries of the knee: 25 years later. Am J Sports Med.

[80] van der List JP, Mintz DN, DiFelice GS (2017). The location of anterior cruciate ligament tears: a prevalence study using magnetic resonance imaging. Orthop J Sports Med.

[81] Frobell RB, Lohmander LS, Roos EM (2007). The challenge of recruiting patients with anterior cruciate ligament injury of the knee into a randomized clinical trial comparing surgical and non-surgical treatment. Contemp Clin Trials.

[82] van Eck CF, Limpisvasti O, ElAttrache NS (2017). Is there a role for internal bracing and repair of the anterior cruciate ligament? A systematic literature review. Am J Sports Med.

[83] Descamps P, Silbert H (2016). Ligamys technique versus standard technique for the anterior cruciate ligament rupture.

[84] Heusdens CH (2018). Single-blind, multi-centre, randomized controlled trial comparing ligamys Anterior Cruciate Ligament (ACL) repair, internal bracing acl repair and conventional acl reconstruction for relative clinical efficacy and economic benefit.

[85] Murray MM, Yeng YM (2016). A prospective, randomized, controlled, clinical trial evaluating the non-inferiority of Bridge-Enhanced Anterior Cruciate Ligament repair (BEAR) to Anterior Cruciate Ligament Reconstruction with an autologous tendon graft (ACLR).

[86] Evangelopoulos DS, Kohl S, Schwienbacher S, Gantenbein B, Exadaktylos A, Ahmad SS (2017). Collagen application reduces complication rates of mid-substance ACL tears treated with dynamic intraligamentary stabilization. Knee Surg Sports Traumatol Arthrosc.

[87] Krismer AM, Gousopoulos L, Kohl S, Ateschrang A, Kohlhof H, Ahmad SS (2017). Factors influencing the success of anterior cruciate ligament repair with dynamic intraligamentary stabilisation. Knee Surg Sports Traumatol Arthrosc.

[88] Murray MM, Martin SD, Martin TL, Spector M (2000). Histological changes in the human anterior cruciate ligament after rupture. J Bone Joint Surg Am.

[89] van der List JP, Hagemans FJA, Hofstee DJ, Jonkers FJ (2020). The role of patient characteristics on the success of nonoperative treatment of anterior cruciate ligament injuries. Am J Sports Med.

[90] Dabis J, Yasen SK, Foster AJ, Pace JL, Wilson AJ (2020). Paediatric proximal ACL tears managed with direct ACL repair is safe, effective and has excellent short-term outcomes. Knee Surg Sports Traumatol Arthrosc.

[91] Träger D, Pohle K, Tschirner W (1995). Anterior cruciate ligament suture in comparison with plasty. A 5-year follow-up study. Arch Orthop Trauma Surg.

[92] van der List JP, DiFelice GS (2016). Preservation of the anterior cruciate ligament: a treatment algorithm based on tear location and tissue quality. Am J Orthop.

